# Effects of menopausal hormone therapy on ambulatory blood pressure and arterial stiffness in postmenopausal Korean women with grade 1 hypertension: a randomized, placebo-controlled trial

**DOI:** 10.1186/s40885-021-00175-1

**Published:** 2021-09-15

**Authors:** Byung-Koo Yoon, Jidong Sung, Yun-Mi Song, Soo-Min Kim, Kyung-A Son, Jun Hyun Yoo, Sung-Ji Park, Duk-Kyung Kim

**Affiliations:** 1 Department of Obstetrics, Gynecology, and Women’s Health, Samsung Medical Center, Sungkyunkwan University School of Medicine, Seoul, Republic of Korea; 2 Department of Medicine, Samsung Medical Center, Sungkyunkwan University School of Medicine, Seoul, Republic of Korea; 3 Department of Family Medicine, Samsung Medical Center, Sungkyunkwan University School of Medicine, Seoul, Republic of Korea

**Keywords:** Hormone replacement therapy, Estradiol, Progestins, Hypertension, Vascular stiffness

## Abstract

**Background:**

Estrogen therapy in early menopausal women decreases the risk of coronary heart disease and parenteral, but not oral, estrogen is reported to reduce blood pressure (BP). Progestogens are typically added to estrogens to prevent unopposed endometrial stimulation. The effects of progestogen on BP have been less well studied to date. This study was conducted to explore the impacts of micronized progesterone (MP4) combined with percutaneous estradiol gel (PEG) on hemodynamics in postmenopausal Korean women with grade 1 hypertension.

**Methods:**

Fifty-two postmenopausal women (aged 49–75 years) with systolic BP (SBP) of 140–160 mmHg or diastolic BP (DBP) of 90–100 mmHg were randomly assigned for 12 weeks to placebo (*n* = 16), estrogen therapy (ET) (*n* = 19) with PEG (0.1 %, 1 g./d), or estrogen + progestogen therapy (EPT, *n* = 17) with PEG and MP4 (100 mg/d). The primary endpoint was ambulatory BP and the secondary endpoints were arterial stiffness as brachial–ankle pulse-wave velocity (baPWV) and aortic parameters on applanation tonometry.

**Results:**

One woman in the ET group dropped out, so 51 participants were finally analyzed. Outcome measures for ambulatory BP and arterial stiffness were not different between groups. Within-group comparisons showed that EPT significantly decreased daytime heart rate and baPWV: the changes from baseline (mean ± standard deviation) were − 2.5 ± 5.7 bpm (*P* = 0.03) and − 0.6 ± 1.4 m/s (*P* = 0.04), respectively. After adjusting for baseline, linear regression analysis revealed a significant difference in the relationship between baseline and 12-week baPWV among groups (*P* = 0.02). The relationship was significantly different between placebo and ET (*P* = 0.03) and EPT (*P* = 0.01), respectively, but not between ET and EPT. Additionally, pooled results of active treatments disclosed that SBP, DBP, PWV, and augmentation index at the aorta were significantly reduced relative to baseline.

**Conclusions:**

There was no difference in ambulatory BP between ET and EPT in postmenopausal Korean women with grade 1 hypertension. Further, ET and EPT similarly decreased baPWV from baseline as compared with placebo. MP4 might not adversely influence estrogen effects on ambulatory BP and arterial stiffness.

**Trial registration:**

Clinical Research Information Registry, KCT0005405, Registered 22 September 2020 - Retrospectively registered, https://cris.nih.go.kr/cris/search/detailSearch.do?all_type=Y&search_page=L&pageSize=10&page=1&seq=17608&search_lang=E.

**Supplementary Information:**

The online version contains supplementary material available at 10.1186/s40885-021-00175-1.

## Background

Cardiovascular disease (CVD) including coronary heart disease (CHD) and stroke is the number 1 cause of death among women worldwide [1] and hypertension belongs to the group of major risk factors of CVD with the strongest evidence for causation [2]. Based on office blood pressure (BP), hypertension is defined as a systolic BP (SBP) of 140 mmHg or higher and/or diastolic BP (DBP) of 90 mmHg or higher. In the case of grade 1 hypertension with a low risk of CVD, lifestyle modifications including dietary alterations may be sufficient to delay or prevent the need for pharmacological intervention [3].

Ambulatory BP monitoring (ABPM) can identify white-coat and masked hypertension, provides night-time readings, and is a stronger predictor of all-cause and cardiovascular mortality when compared with clinic BP [4]. The loss of arterial elasticity leads to an increase in SBP and a decrease in DBP [3]. Thus, arterial stiffness can precede hypertension and, importantly, be reversible in conjunction with lifestyle change or anti-hypertensive treatment [5].

A sex difference in the prevalence of hypertension is apparent: such is lower in women aged 20 to 34 years than in men but increases steeply after menopause, leading to a cross-over after the age of 70 years [6, 7]. Further, the role of hypertension in death is greater in women than in men [8]. These statistics strongly suggest a key role of endogenous ovarian hormones in hypertension. If initiated in early menopause, menopausal hormone therapy (MHT) using oral estrogen decreases the risk for CHD but has no impact on stroke [9]. This might be attributable to a higher contribution of hypertension to development of stroke than CHD [2]. The effects of estrogen therapy on BP may differ by the route of administration probably via a first-pass hepatic effect. Conjugated equine estrogen (CEE), an oral estrogen, increases the clinic BP [10], whereas parenteral estrogen using a transdermal patch decreases ambulatory BP in postmenopausal women with both hypertension [11] and normal BP [12]. When compared with patch application, percutaneous estradiol gel (PEG), another parenteral preparation, has lower adverse skin effects and could provide higher estradiol serum values with less day-to-day variation [13]. Progestogens are typically added to estrogens to prevent unopposed endometrial stimulation. Medroxyprogesterone acetate (MPA) prescription is on the steep decline these days because of adverse impacts on estrogen in CHD [14] and breast cancer [15]. Thus, the search for a safe and effective progestogen for MHT is one of the top-priority matters in the field of menopause. The effects of progestogen on BP have been less well studied to date. Our group [16] previously reported that micronized progesterone (MP4), combined with CEE, exerted favorable impacts on ambulatory BP in a prospective study.

The purpose of this study was to evaluate the impact of MP4 added to PEG on ambulatory BP and arterial stiffness in postmenopausal Korean women with grade 1 hypertension.

## Methods

### Study participants

A total of 61 postmenopausal women aged 57.3 ± 4.9 years (range: 49–75 years) with grade 1 hypertension were enrolled in this study. Women were considered postmenopausal if their duration of amenorrhea was at least 12 months or a serum follicle-stimulating hormone (FSH) value of 30 mIU/mL or higher was recorded. According to clinic BP measured at the arm, grade 1 hypertension was defined as an SBP of 140 to 159 mmHg or a DBP of 90 to 99 mmHg. Women with uncontrolled hypertension using antihypertensive medication for at least six months were also enrolled and asked to maintain their current BP medication. In addition, women whose BP was controlled but who wanted to hold their BP medication were included if the BP rebounded only to grade 1 after two weeks of washout. Patients with symptomatic coronary heart disease or stroke were not eligible. Women were also excluded if they had current or recent (within one year prior to enrollment) smoking; uncontrolled diabetes mellitus (glycated hemoglobin > 8 %); secondary hypertension; hypertension with serious target organ injury suspected; current or recent (within three months before the time of the study entry) MHT use; or contraindications for MHT including acutely impaired liver function, breast cancer, and venous thrombosis. Informed consent was obtained from the participant and the current study was approved by the Institutional Review Board of the hospital (IRB No: 2010-12-019-001).

### Study design

We conducted a prospective, randomized, double-blind, placebo-controlled trial that spanned 12 weeks from November 2011 to January 2017. Participants were randomly allocated in a 1:1:1 ratio to placebo (*n* = 16), estrogen therapy (ET) (*n* = 19), or estrogen + progestogen therapy (EPT) (*n* = 17). The allocation of treatment was based on randomization codes created by the SAS program (SAS Institute, Cary, NC, USA). No other specific randomization stratification factor was applied. PEG (0.1 %, 1 g./d; Samil Pharm. Co., Seoul, Korea) was applied to the forearm before sleep. For EPT, oral MP4 (100 mg/d; Besins Healthcare, Brussel, Belgium) was added to PEG. Two kinds of placebo identical in appearance to the active treatment drug were kindly supplied by each drug company.

Clinic BPs were followed up with at four and 12 weeks. Participants whose BP increased during the study to grade 2 or higher were removed from the study population and closely followed up with by an internist.

The primary and secondary endpoints of the current study were ambulatory BPs and arterial stiffness, respectively.

### Outcome measures

ABPM was performed for a 24-hour period at baseline and 12 weeks after MHT using an automated portable device (Suntech Medical Instruments Inc., Raleigh, NC, USA), which used the oscillometric method and R-wave gating to measure BP, as previously described [16]. Briefly, the mean SBP and DBP values for the daytime (07.00–22.00) and nighttime (22.00–07.00) periods were calculated as the mean values of the hourly averages. The nightly dip in BP was determined as the difference between the daytime and nighttime BPs. The BP load was calculated as the percentage of BP that was higher than predefined values (135/85 for the daytime period and 130/80 for the nighttime period).

Arterial stiffness was evaluated with both pulse-wave velocity (PWV) using the incident wave and augmentation index (AIx) using the reflected wave. Brachial–ankle PWV (baPWV) (VP-1000; Colin, Komaki, Japan) measurements were made after at least five minutes of rest. The volume-rendering method was used in PWV determination. The right- and left-side baPWVs were measured and the average of the bilateral baPWV values was adopted into the analysis. Blood pressures were obtained from all four limbs simultaneously using the oscillometric method. The ankle–brachial index (ABI) was calculated. The validity and reproducibility of this method have been previously reported [17]. The peripheral pulse pressure curve was recorded at the radial artery by means of applanation tonometry (SphygmoCor; Atcor Medical, Sydney, Australia). The SphygmoCor personal computer software calculates aortic SBP and aortic pulse pressure (PP) (i.e., the difference between SBP and DBP) by the transformation of the radial pulse wave. The inflection point was identified within the time domain, indicating the arrival of the reflected wave in the ascending aorta. The BP at this point in time is the inflection pressure, while the difference between aortic SBP and inflection pressure is augmentation pressure (AP). The AIx was then calculated by AP/aortic PP × 100 [18].

### Hormone assay

Blood concentrations of FSH, estradiol, and progesterone were measured at baseline and 12 weeks of treatment, using radioimmunoassay (DIA source, Belgium for FSH; Beckman Coulter, Czech for estradiol and progesterone).

### Statistical analysis

Statistical analyses were performed using SAS version 9.4. Data are expressed as means ± standard deviations or numbers (%). Baseline characteristics between the three study groups were compared using the Kruskal Wallis test for continuous variables or Fisher’s exact test for categorical variables. Between-group comparisons of the outcome measures were examined with the Kruskal–Wallis test for three groups or the Wilcoxon rank-sum test for two groups and within-group comparisons were examined with the signed-rank test. The difference of 12-week values among the three groups was analyzed with adjustment for baseline values using linear regression analysis. For all analyses, a two-tailed *P*-value < 0.05 was considered to be statistically significant.

## Results

**Fig. 1:**
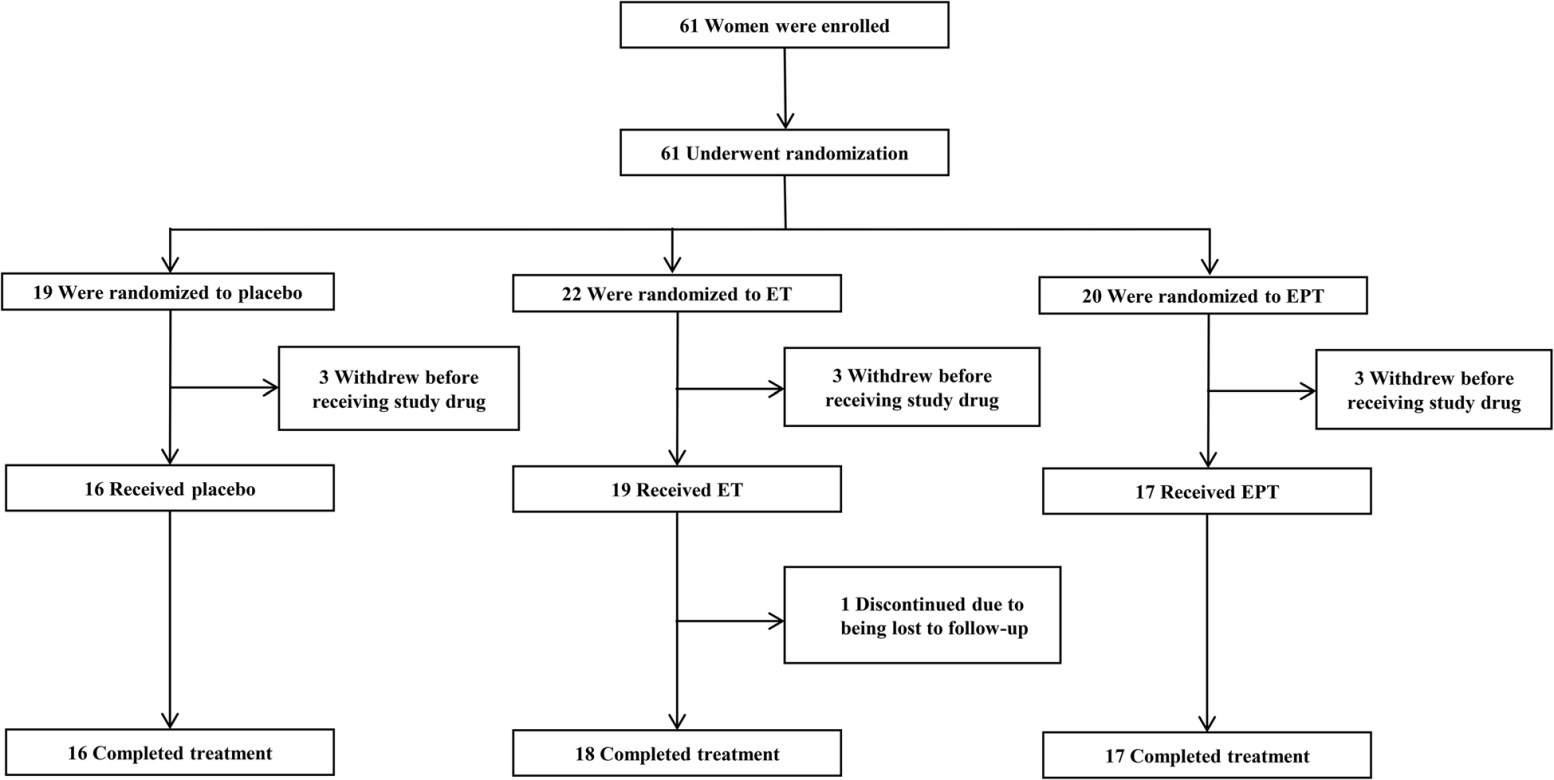
Flow diagram of the study

The flow diagram for the study is displayed in Fig. 1. Sixty-one women took part in this trial and three women decided to withdraw before treatment in each group. As a result, a total of 52 received the study drug, including 16 placebo cases, 19 ET cases, and 17 EPT cases. Overall, only one woman in the ET group dropped out due to being lost to follow-up, so 51 participants were finally analyzed.

Baseline characteristics of the study participants are summarized in Table 1. All variables except a medical history of dyslipidemia were comparable among the three groups. Table 2 shows blood hormone concentrations: there was no difference in FSH, estradiol, and progesterone at baseline between the three groups, but significant group difference was found at 12 weeks in all three hormones.

**Table. 1:** Baseline characteristics of study participants

	Placebo(*n* = 16)	ET(*n* = 18)	EPT(*n* = 17)
Age (years)	56.6 ± 3.8	57.7 ± 6.3	57.5 ± 4.4
Age at menarche (years)	14.8 ± 1.1	14.9 ± 2.4	15.1 ± 1.7
Type of menopause			
Natural	15 (92.7)	16 (88.9)	16 (94.1)
Surgical	1 (6.3)	2 (11.1)	1 (5.9)
Age at menopause (years)	52.2 ± 2.8	49.8 ± 4.8	51.2 ± 3.9
Duration from menopause (months)	39.6 ± 31.1	62.9 ± 41.4	63.9 ± 61.6
Parity	1.9 ± 0.9	2.1 ± 0.4	2.2 ± 0.5
Body mass index (kg/m^2^)	23.5 ± 1.7	24.6 ± 2.9	24.9 ± 3.2
Medical history			
Diabetes mellitus	1 (6.25)	0 (0.0)	1 (5.9)
Dyslipidemia^a^ [statin use]	2 (12.5) [2]	8 (44.4) [3]	2 (11.8) [2]
Antihypertensive medication use	11 (68.8)	11 (61.1)	8 (47.1)
History of hormone use			
Oral contraceptive	2 (12.5)	1 (5.6)	0 (0.0)
Menopausal hormone therapy	4 (25.0)	7 (38.9)	5 (29.4)
Current smoking	0 (0.0)	1 (5.6)	0 (0.0)
Alcohol consumption	1 (6.3)	3 (16.7)	4 (23.5)
Family history of hypertension	7 (43.8)	13 (72.2)	8 (47.1)

Data are presented as means ± standard deviations or numbers (%)

ET estrogen therapy, EPT estrogen + progestogen therapy

^a^*P*-value for between-group differences < 0.05, by Fisher’s exact test

**Table. 2:** Blood hormone levels in study participants with menopausal hormone therapy

	Placebo (*n* = 16)			ET (*n* = 18)			EPT (*n* = 17)		
	Baseline	12 weeks	Change from baseline	Baseline	12 weeks	Change from baseline	Baseline	12 weeks	Change from baseline
FSH^a^	54.6 ± 17.6	53.7 ± 17.2	-0.3 ± 16.9	43.2 ± 14.3	29.5 ± 16.1	-14.5 ± 15.9^b^	44.1 ± 22.7	24.8 ± 12.1	-20.4 ± 19.4^b^
E2^a^	15.5 ± 16.8	42.0 ± 48.0	23.2 ± 56.3	16.4 ± 16.7	119.7 ± 115.4	104.0 ± 112.6^b^	19.8 ± 32.4	68.3 ± 43.2	56.1 ± 46.6^b^
P4^a^	0.2 ± 0.1	0.1 ± 0.1	-0.1 ± 0.1	0.1 ± 0.2	0.1 ± 0.0	-0.1 ± 0.2	0.2 ± 0.3	2.8 ± 2.1	2.5 ± 2.1^b^

Data are presented as means ± standard deviations

ET estrogen therapy, EPT estrogen + progestogen therapy, FSH follicle stimulating hormone (mIU/mL), E2 estradiol (pg/ml), P4 progesterone (ng/ml)

^a^*P*-value for between-group difference at 12 weeks of treatment < 0.05, by the Kruskal Wallis test

^b^*P*-value for within-group difference between baseline and 12 weeks of treatment < 0.05, by the signed-rank test

No participant discontinued the study because of a BP increase to grade 2 hypertension or higher. The effects of MHT on ambulatory BPs are summarized in Table 3. There was no difference in BP measurements between the three groups. Changes from baseline were also comparable between the three groups. The changes from baseline in 24-hour SBP in the placebo, ET, and EPT groups were − 1.5 ± 11.7, -1.0 ± 13.4, and − 1.5 ± 9.3 mmHg, respectively (*P* = 0.85); those in 24-hour DBP were − 2.5 ± 7.9, -0.3 ± 6.1, and − 1.3 ± 5.4 mmHg, respectively (*P* = 0.38). Relative to baseline, the daytime heart rate (HR) was significantly decreased in the EPT group (change from baseline; -2.5 ± 5.7bpm, *P* = 0.03). The pooled results of the ET and EPT groups did not suggest a difference in any ambulatory BP as compared with either the placebo group or baseline (see Additional file  1).

**Table. 3:** Effect of menopausal hormone therapy on ambulatory blood pressure measures

	Placebo (*n* = 16)			ET (*n* = 18)			EPT (*n* = 17)		
	Baseline	12 weeks	Change from baseline	Baseline	12 weeks	Change from baseline	Baseline	12 weeks	Change from baseline
Daytime measurement									
SBP	147.9 ± 14.3	146.9 ± 11.0	-0.9 ± 12.5	148.0 ± 14.5	146.7 ± 20.0	-1.3 ± 14.2	150.0 ± 11.6	148.5 ± 11.6	-1.4 ± 9.5
DBP	92.8 ± 9.4	91.0 ± 10.6	-2.3 ± 8.7	93.5 ± 9.9	93.8 ± 11.4	0.3 ± 7.1	94.5 ± 10.4	93.3 ± 10.1	-1.2 ± 6.1
HR	72.8 ± 6.8	72.0 ± 7.4	0.1 ± 6.4	73.7 ± 6.3	72.2 ± 7.0	-1.5 ± 6.3	76.6 ± 7.8	74.1 ± 7.4	-2.5 ± 5.7 ^c^
BP load^a^									
SBP	77.0 ± 26.0	74.1 ± 25.5	-2.4 ± 28.2	73.6 ± 26.8	65.8 ± 31.2	-7.8 ± 29.0	77.4 ± 22.8	75.2 ± 20.5	-2.2 ± 16.8
DBP	74.3 ± 25.5	66.2 ± 30.9	-10.5 ± 23.7	66.3 ± 23.6	66.6 ± 27.9	0.2 ± 24.6	71.2 ± 27.2	69.8 ± 31.0	-1.4 ± 10.5
Nighttime measurement									
SBP	139.9 ± 16.8	136.4 ± 12.6	-3.7 ± 12.4	135.1 ± 13.6	135.9 ± 17.3	0.8 ± 15.9	145.2 ± 12.1	143.9 ± 12.6	-1.3 ± 10.7
DBP	85.3 ± 11.5	82.4 ± 9.4	-3.2 ± 7.8	83.5 ± 11.3	82.1 ± 9.8	-1.4 ± 8.8	89.1 ± 10.0	87.7 ± 10.0	-1.4 ± 4.3
HR	64.0 ± 6.4	62.2 ± 6.6	-1.2 ± 7.8	61.4 ± 6.3	61.6 ± 4.9	0.2 ± 3.4	66.2 ± 6.6	62.7 ± 5.9	-3.5 ± 7.1
BP load^a^									
SBP	79.1 ± 25.9	77.8 ± 28.5	-2.2 ± 32.1	70.7 ± 28.3	67.2 ± 31.8	-3.5 ± 40.4	89.4 ± 17.8	82.3 ± 26.1	-7.1 ± 21.1
DBP	59.9 ± 27.9	49.4 ± 32.6	-10.8 ± 28.3	56.3 ± 35.3	51.8 ± 34.6	-4.5 ± 39.3	67.3 ± 33.4	61.4 ± 37.9	-5.9 ± 24.3
Night dip^b^									
SBP	-5.3 ± 7.4	-7.8 ± 7.0	-2.7 ± 6.3	-8.3 ± 8.9	-7.1 ± 4.1	1.2 ± 8.2	-2.9 ± 7.9	-3.0 ± 6.9	-0.1 ± 5.2
DBP	-8.0 ± 7.9	-9.6 ± 8.0	-1.4 ± 7.5	-10.3 ± 10.8	-12.0 ± 8.7	-1.7 ± 10.2	-5.4 ± 7.6	-5.8 ± 7.6	-0.4 ± 5.0
24-hour measurement									
SBP	146.0 ± 14.1	144.4 ± 10.5	-1.5 ± 11.7	144.8 ± 13.0	143.8 ± 19.2	-1.0 ± 13.4	148.8 ± 10.6	147.3 ± 10.9	-1.5 ± 9.3
DBP	91.0 ± 9.4	89.0 ± 9.9	-2.5 ± 7.9	91.2 ± 9.3	90.8 ± 10.4	-0.3 ± 6.1	93.2 ± 9.7	91.9 ± 9.5	-1.3 ± 5.4

Data are presented as means ± standard deviations

ET estrogen therapy, EPT estrogen + progestogen therapy, SBP systolic blood pressure (mmHg), DBP diastolic blood pressure (mmHg), HR heart rate (bpm), BP blood pressure

^a^ proportion (%) of BP higher than the predefined cutoff level (135/85 mmHg for the daytime and 130/80 mmHg for the nighttime)

^b^BP difference (mmHg) between the daytime and nighttime

^c^*P*-value for within-group difference between baseline and 12 weeks of treatment < 0.05, by the signed-rank test

**Fig. 2:**
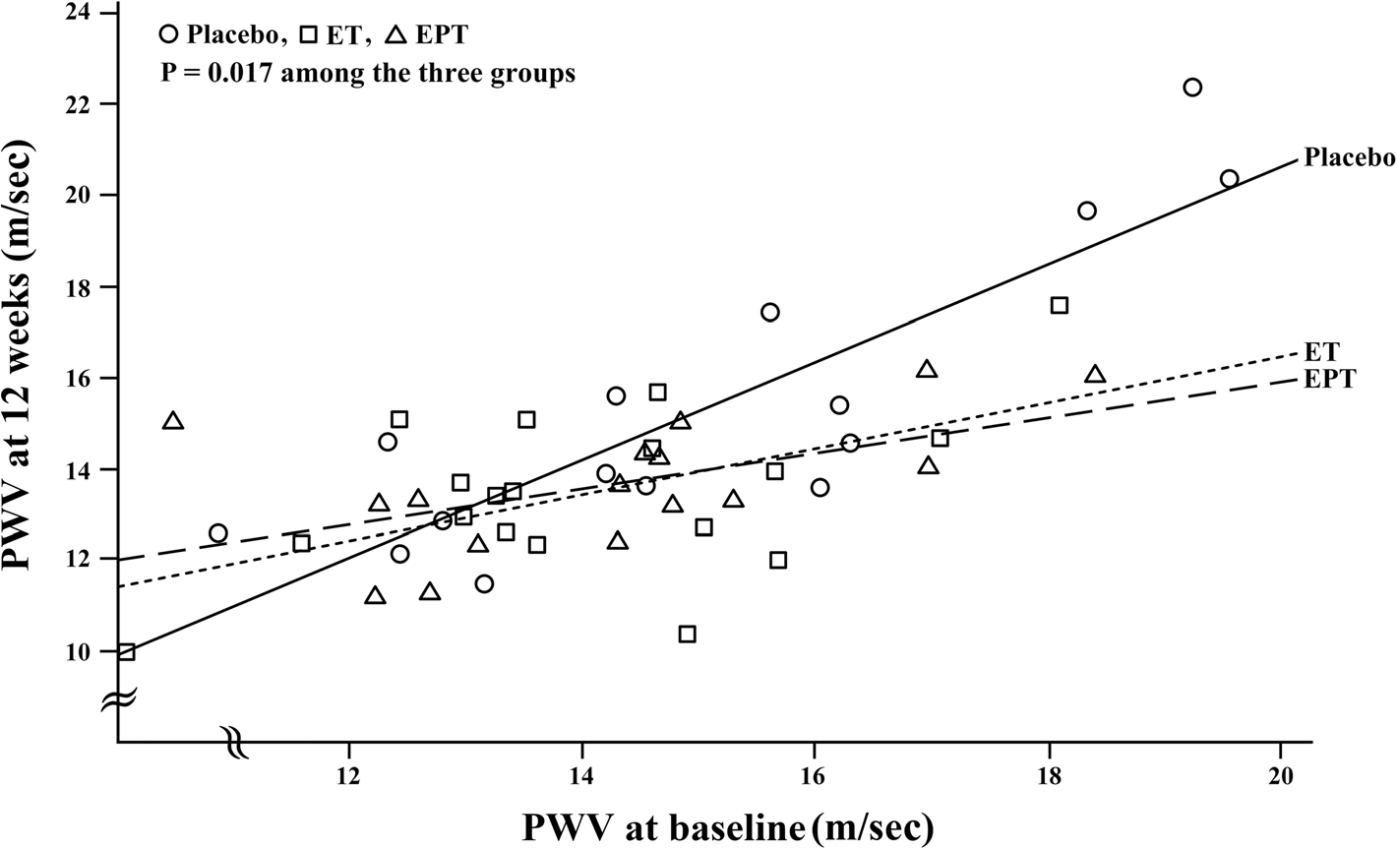
The relationship between baseline and 12-week measurements of brachial-ankle pulse-wave velocity (PWV). ET, estrogen therapy; EPT, estrogen + progestogen therapy. The beta value (standard error) was − 0.61 (0.27) for the ET group (*P* = 0.03) and − 0.74 (0.27) for the EPT group (*P* = 0.01), examined with linear regression analysis after adjusting for the baseline

**Fig. 3:**
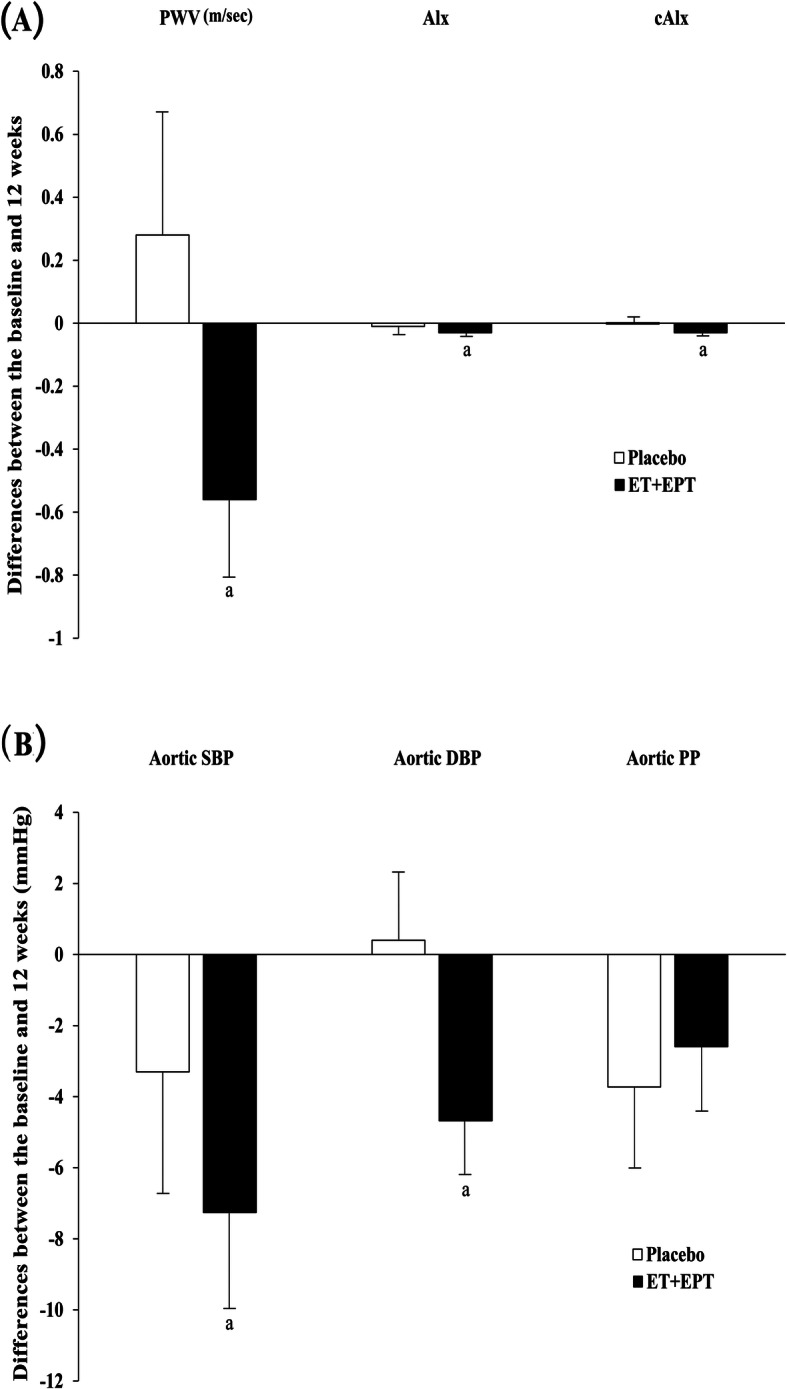
Mean differences between baseline and 12 weeks in the placebo and pooled active treatment groups. (a) Arterial stiffness. (b) Central blood pressure (BP). ET, estrogen therapy; EPT, estrogen + progestogen therapy; PWV, brachial–ankle pulse-wave velocity; AIx, augmentation index; cAIx, corrected augmentation index at a heart rate of 75 bpm; SBP, systolic BP; DBP, diastolic BP; PP, pulse pressure; I bars indicate standard errors. There was no significant difference between groups. ^a^*P*-value for within-group difference between baseline and 12 weeks of treatment < 0.05, by the signed -rank test

There was no participant with a baseline ABI measurement of 0.9 or less, which is highly suggestive of peripheral arterial disease. As shown in Table 4, the main markers of arterial stiffness did not differ between the three groups. Within the EPT group, PWV significantly fell after 12 weeks as compared with baseline (change from baseline; -0.6 ± 1.4m/s, *P* = 0.04). After controlling for baseline value, the linear regression analysis of PWV disclosed that the relationship between baseline and 12-week values was significantly different among the three groups (*P* = 0.02) (Fig. 2). The relationship was significantly different between the placebo and ET groups (beta value = -0.61, standard error = 0.27; *P* = 0.03) and between the placebo and EPT groups (beta value = -0.74, standard error = 0.27; *P* = 0.01) but was comparable between the ET and EPT groups, meaning that the ET and EPT groups showed a significantly greater decrease in PWV from baseline relative to the placebo group. As compared with baseline, the aortic DBP was significantly lowered after 12 weeks of ET (*P* = 0.03). Further, AIx, but not corrected AIx, was significantly diminished within the ET group (*P* = 0.03). When ET and EPT were combined, between-group comparisons showed that differences between at baseline and 12 weeks did not differ. However, within-group analysis revealed that the active treatments decreased the PWV (*P* = 0.02), AIx (*P* = 0.02), and corrected AIx at a HR of 75bpm (*P* = 0.01) (Fig. 3A). In addition, the pooled results suggested significant reductions in aortic SBP (*P* = 0.02) and DBP (*P* = 0.00) relative to baseline (Fig. 3B)

**Table. 4:** Effects of menopausal hormone therapy on arterial stiffness

	Placebo (*n* = 16)			ET (*n* = 18)			EPT (*n* = 17)		
	Baseline	12 weeks	Change from baseline	Baseline	12 weeks	Change from baseline	Baseline	12 weeks	Change from baseline
PWV	15.8 ± 2.2	16.3 ± 2.9	0.3 ± 1.5	15.2 ± 1.6	14.6 ± 1.7	-0.6 ± 1.6	15.4 ± 1.7	14.9 ± 1.3	-0.6 ± 1.4^a^
Ao SBP	137.3 ± 14.2	134.6 ± 13.7	-3.3 ± 13.8	135.9 ± 10.6	128.1 ± 18.3	-7.9 ± 15.3	140.9 ± 14.6	136.7 ± 16.2	-6.6 ± 17.0
Ao DBP	85.2 ± 10.5	86.3 ± 9.9	0.4 ± 7.5	87.7 ± 6.0	82.1 ± 10.3	-5.6 ± 9.1^a^	90.1 ± 9.0	87.9 ± 10.0	-3.6 ± 8.2
Ao PP	52.1 ± 7.7	48.3 ± 7.4	-3.7 ± 8.6	48.3 ± 9.7	46.0 ± 13.4	-2.3 ± 10.1	50.8 ± 11.3	48.8 ± 12.0	-2.9 ± 11.6
AIx	0.40 ± 0.09	0.39 ± 0.09	0.0 ± 0.1	0.39 ± 0.05	0.35 ± 0.07	0.0 ± 0.1^a^	0.40 ± 0.09	0.39 ± 0.09	0.0 ± 0.1
cAIx	0.35 ± 0.07	0.34 ± 0.06	0.0 ± 0.1	0.33 ± 0.05	0.34 ± 0.06	0.0 ± 0.1	0.35 ± 0.08	0.33 ± 0.07	0.0 ± 0.1

Data are presented as means ± standard deviations

ET estrogen therapy, EPT estrogen + progestogen therapy, Ao aortic, SBP systolic blood pressure (mmHg), DBP diastolic blood pressure (mmHg), PP pulse pressure (mmHg), PWV brachial–ankle pulse-wave velocity (m/s), AIx augmentation index, cAIx corrected augmentation index at heart rate 75 bpm

^a^*P*-value for within-group difference between baseline and 12 weeks of treatment < 0.05, by the signed-rank test

The placebo group reported no adverse effect, whereas three participants (16.7 %) in the ET group and seven (41.2 %) in the EPT group experienced adverse effects including vaginal bleeding and breast change most frequently.

## Discussion

The present study was carried out to examine the impacts of MP4 combined with PEG in postmenopausal Korean women with grade 1 hypertension. The main finding was that there was no difference in ambulatory BP between ET and EPT. Further, ET and EPT comparably decreased the PWV from baseline as compared with the placebo. MP4 might not adversely influence the estrogen effect on ambulatory BP and arterial stiffness.

As compared with CEE, adopting a transdermal route for estrogen showed a lower risk of venous thrombosis [19] and stroke [20]. MP4 is also considered safer than MPA in terms of CVD, venous thrombosis, and breast cancer [21]. Accordingly, PEG/MP4 may be a favorable regimen of MHT for postmenopausal women with hypertension.

The current study reported that the results of ABPM were comparable among the three groups overall. The estradiol patch was reported previously to decrease BP in hypertensive postmenopausal women [11]; the study authors found that a higher (100 µg/d) patch dose over the conventional (50 µg/d) dose significantly reduced both SBP and DBP during a 24-hour and daytime periods in postmenopausal women with untreated, mild to moderate hypertension. In the current study, however, neither SBP nor DBP were changed with ET. Previously, our group [16] found a significant negative correlation between baseline BP and BP change with MHT. The current study recruited women with grade 1 hypertension due to potential ethical issue and a lower dose of PEG (1 g./d) was given relative to the conventional dose (1.5 g./d) to explore the MP4 effects better. Additionally, the proportion of participants with untreated hypertension was lower. These differences might account, at least partly, for the negative result on ABPM.

Carotid–femoral PWV (cfPWV) is the gold standard for assessing aortic stiffness [22], which is measured by tonometry or Doppler and requires specialized training and exposure of the inguinal region. baPWV is a unique measure of systemic arterial stiffness applied using an oscillometric method, which is easy and reproducible and which is closely correlated with cfPWV [23]. This study showed that baPWV did not differ between the three groups initially. Compared with baseline, however, baPWV was declined with EPT. After controlling for the baseline value, regression analysis further revealed a statistically significant difference in the relationship between baseline and 12-week baPWV values among the three groups. Both the outcomes of the ET and EPT groups were similar to each other and different from the placebo, which suggests that MP4 does not alter the beneficial estrogen effect on baPWV. Arterial stiffness accelerates in the early postmenopausal phase and this might be related to estrogen deficiency [24], but the results of randomized controlled trials considering the estrogen effect on PWV differ by the presence of hypertension and route of administration. In normotensive women, estrogen therapy did not affect PWV regardless of the route of administration [25, 26]. In hypertensive women, an estrogen patch [27] but not oral estrogen [28] improved PWV independent of BP change, which agrees with the current study. Even though the mechanism is not fully understood, estrogen might improve arterial stiffness through vasodilation [16] and restoration of the aberrant vascular matrix [29]. Regarding progestogen, drospirenone, an antimineralocorticoid progestin, decreased PWV in normotensive women [30]. To our knowledge, this is the first study to report the effect of natural progesterone on arterial stiffness in women with hypertension. Further research is needed to clarify the underlying mechanism.

Aortic BPs and PP can be calculated by transformation of the radial pulse wave, and AIx is considered as a marker of aortic stiffness. Central BPs and AIx were not different between the three groups, but within-group analysis revealed decreases in the aortic DBP and AIx in the ET group. When combined, favorable impacts of active treatments on central aortic BPs and arterial stiffness became evident, likely due to enhanced statistical power. Despite a paucity of literature, MP4 might have independent action on BP, too. MP4 shows antimineralocorticoid effects and lowers the pressure response to angiotensin II. In addition, MP4 increases nitric oxide production, suppresses vasoconstriction by the modulation of calcium channels, and decreases vascular resistance [16]. These findings at minimum exclude the adverse impact of MP4 on estrogen action. The aortic PP did not change probably because of the decreases in both SBP and DBP.

Of note, the current study reports that combined results of ET and EPT showed a decrease in central BPs but not peripheral BPs in postmenopausal women with grade 1 hypertension. The aortic SBP is actually lower than the brachial measurement, although this difference is highly variable between individuals. Furthermore, some studies suggested that central pressure is better related to future cardiovascular events as compared with brachial BP [31]. Likewise, a differential impact of BP-lowering drugs on central versus peripheral pressures was reported and aortic SBP and PP may be determinants of clinical outcomes [32]. This study also suggested that MHT might prevent an increase in central PP, probably by improving aortic stiffness. In addition, an increase in central BP may predate that in peripheral BP during the early phase of hypertension development [33]. Therefore, longer-term treatment might yield a reduction in peripheral BP later on.

In this study, the proportion of women with untreated hypertension was too low to study the MHT effect fully. Moreover, the sample size was small and the follow-up period was short. Additional large-scale, long-term trials are required to confirm our findings. We studied the effects of PEG and MP4 in postmenopausal women with grade 1 hypertension and the results of this study cannot be generalized to a higher degree of hypertension or other regimens of MHT.

Our study has several strengths worth mentioning. This was a randomized, double-blinded trial performed with separate arms for estrogen alone and the combination of estrogen and progestogen, respectively. Up-to-date technologies were applied to evaluate the MHT effect on hemodynamics. Impacts on central BP and PP as well as peripheral BP during a 24-hour period were assessed. In addition, both central and systemic arterial stiffness were examined. Nonetheless, the conclusions of this study are preliminary due to the limitations mentioned above. Further studies are warranted.

## Conclusions

Our study suggests that MP4 does not negate estrogen effects on ambulatory BP and arterial stiffness in postmenopausal Korean women with grade 1 hypertension.

## Supplementary information

**Additional file 1.**

## Data Availability

The datasets used and/or analyzed during the current study are available from the corresponding author on reasonable request.

## Abbreviations

ABI: Ankle–brachial index
ABPM: Ambulatory blood pressure monitoring
AIx: Augmentation index
AP: Augmentation pressure
baPWV: brachial–ankle pulse-wave velocity
BP: Blood pressure
CEE: Conjugated equine estrogen
cfPWV: carotid–femoral PWV
CHD: Coronary heart disease
CVD: Cardiovascular disease
DBP: Diastolic blood pressure
ET: Estrogen therapy
EPT: Estrogen + progestogen therapy
FSH: Follicle-stimulating hormone
HR: Heart rate
MHT: Menopausal hormone therapy
MPA: Medroxyprogesterone acetate
MP4: Micronized progesterone
PEG: Percutaneous estradiol gel
PP: Pulse pressure
PWV: Pulse-wave velocity
SBP: Systolic blood pressure

## References

[1] Roth GA, Johnson C, Abajobir A, Abd-Allah F, Abera SF, Abyu G (2017). Global, Regional, and National Burden of Cardiovascular Diseases for 10 Causes, 1990 to 2015. J Am Coll Cardiol.

[2] Fuchs FD, Whelton PK (2020). High blood pressure and cardiovascular disease. Hypertension.

[3] Williams B, Mancia G, Spiering W, Agabiti Rosei E, Azizi M, Burnier M (2018). 2018 ESC/ESH Guidelines for the management of arterial hypertension. Eur Heart J.

[4] Yang WY, Melgarejo JD, Thjs L, Zhang ZY, Boggia J, Wei FF (2019). Association of office and ambulatory blood pressure with mortality and cardiovascular outcomes. JAMA.

[5] Oh YS (2018). Arterial stiffness and hypertension. Clin Hypertens.

[6] Benjamin EJ, Virani SS, Callaway CW, Chamberlain AM, Chang AR, Cheng S (2018). Heart disease and stroke statistics-2018 update: a report from the American Heart Association. Circulation.

[7] The Korean Statistical Information Service. http://kosis.kr/statHtml/statHtml.do?orgId=117&tblId=DT_11702_N105. Accessed 15 Feb. 2020.

[8] Abramson BL, Melvin RG (2014). Cardiovascular risk in women: focus on hypertension. Can J Cardiol.

[9] Boardman HM, Hartley L, Eisinga A, Main C, Roqué i Figuls M, Bonfill Cosp X (2015). Hormone therapy for preventing cardiovascular disease in post-menopausal women. Cochrane Database Syst Rev.

[10] Shimbo D, Wang L, Lamonte MJ, Allison M, Wellenius GA, Bavry AA (2014). The effect of hormone therapy on mean blood pressure and visit-to-visit blood pressure variability in postmenopausal women: results from the Women’s Health Initiative randomized controlled trials. J Hypertens.

[11] Mercuro G, Zoncu S, Pilia I, Lao A, Melis GB, Cherchi A (1997). Effects of acute administration of transdermal estrogen on postmenopausal women with systemic hypertension. Am J Cardiol.

[12] Zacharieva S, Atanassova I, Kirilov G, Kalinov K, Shigarminova R, Nachev E (2002). Effect of transdermal estrogen therapy on some vasoactive humoral factors and 24-h ambulatory blood pressure in normotensive postmenopausal women. Climacteric.

[13] Scott RT, Ross B, Anderson C, Archer DF (1991). Pharmacokinetics of percutaneous estradiol: a crossover study using a gel and a transdermal system in comparison with oral micronized estradiol. Obstet Gynecol.

[14] Rossouw JE, Prentice RL, Manson JE, Wu L, Barad D, Barnabei VM (2007). Postmenopausal hormone therapy and risk of cardiovascular disease by age and years since menopause. JAMA.

[15] Manson JE, Chlebowski RT, Stefanick ML, Aragaki AK, Rossouw JE, Prentice RL (2013). Menopausal hormone therapy and health outcomes during the intervention and extended poststopping phases of the Women’s Health Initiative randomized trials. JAMA.

[16] Lee DY, Kim JY, Kim JH, Choi DS, Kim DK, Koh KK (2011). Effects of hormone therapy on ambulatory blood pressure in postmenopausal Korean women. Climacteric.

[17] Yamashina A, Tomiyama H, Takeda K, Tsuda H, Arai T, Hirose K (2002). Validity, reproducibility, and clinical significance of noninvasive brachial-ankle pulse wave velocity measurement. Hypertens Res.

[18] Crilly M, Coch C, Bruce M, Clark H, Williams D (2007). Indices of cardiovascular function derived from peripheral pulse wave analysis using radial applanation tonometry: a measurement repeatability study. Vasc Med.

[19] Laliberté F, Dea K, Duh MS, Kahler KH, Rolli M, Lefebvre P (2011). Does the route of administration for estrogen hormone therapy impact the risk of venous thromboembolism? Estradiol transdermal system versus oral estrogen-only hormone therapy. Menopause.

[20] Renoux C, Dell’aniello S, Garbe E, Suissa S (2010). Transdermal and oral hormone replacement therapy and the risk of stroke: a nested case-control study. BMJ.

[21] L’Hermite M (2013). HRT optimization, using transdermal estradiol plus micronized progesterone, a safer HRT. Climacteric.

[22] Safar ME, Asmar R, Benetos A, Blacher J, Boutouyrie P, Lacolley P (2018). Interaction between hypertension and arterial stiffness. Hypertension.

[23] Ohkuma T, Ninomiya T, Tomiyama H, Kario K, Hoshide S, Kita Y (2017). Brachial-ankle pulse wave velocity and the risk prediction of cardiovascular disease: an individual participant data meta-analysis. Hypertension.

[24] Zaydun G, Tomiyama H, Hashimoto H, Arai T, Koji Y, Yambe M (2006). Menopause is an independent factor augmenting the age-related increase in arterial stiffness in the early postmenopausal phase. Atherosclerosis.

[25] Dias AR, de Mello NR, Eluf Gebara OC, Nussbacher A, Wajngarten M, Petti DA (2008). Conjugated equine estrogen, raloxifene and arterial stiffness in postmenopausal women. Climacteric.

[26] Teede HJ, Liang YL, Kotsopoulos D, Zoungas S, Craven R, McGrath BP (2002). Placebo-controlled trial of transdermal estrogen therapy alone in postmenopausal women: effects on arterial compliance and endothelial function. Climacteric.

[27] da Costa LS, de Oliveira MA, Rubim VS, Wajngarten M, Aldrighi JM, Rosano GM (2004). Effects of hormone replacement therapy or raloxifene on ambulatory blood pressure and arterial stiffness in treated hypertensive postmenopausal women. Am J Cardiol.

[28] Arruda CG, Aldrighi JM, Bortolotto LA, Alecrin IN, Ramires JA (2006). Effects of estradiol alone and combined with norethisterone acetate on pulse-wave velocity in hypertensive postmenopausal women. Gynecol Endocrinol.

[29] Fischer GM (1972). In vivo effects of estradiol on collagen and elastin dynamics in rat aorta. Endocrinology.

[30] Vitale C, Mammi C, Gambacciani M, Russo N, Spoletini I, Fini M (2017). Effect of hormone replacement therapy with the anti-mineralocorticoid progestin Drospirenone compared to tibolone on endothelial function and central haemodynamics in post-menopausal women. Int J Cardiol.

[31] Ochoa A, Patarroyo-Aponte G, Rahman M (2018). The role of central blood pressure monitoring in the management of hypertension. Curr Cardiol Rep.

[32] Williams B, Lacy PS, Thom SM, Cruickshank K, Stanton A, Collier D (2006). Differential impact of blood pressure-lowering drugs on central aortic pressure and clinical outcomes: principal results of the Conduit Artery Function Evaluation (CAFE) study. Circulation.

[33] Harvey RE, Johnson MC, Ranadive SM, Joyner MJ, Lahr BD, Miller VM (2017). Aortic hemodynamics in postmenopausal women following cessation of hormone therapy. Physiol Rep.

