# Supplementary material for: Effects of menopausal hormone therapy on ambulatory blood pressure and arterial stiffness in postmenopausal Korean women with grade 1 hypertension: a randomized, placebo-controlled trial
Source: Clin Hypertens. 2021 Sep 15;27:18. doi: 10.1186/s40885-021-00175-1 (PMC8442403; doi:10.1186/s40885-021-00175-1)
Supplement: Supplementary file 1 — Additional file 1. [file 40885_2021_175_MOESM1_ESM.docx]

**Table S** Effect of menopausal hormone therapy on ambulatory blood pressure measures: pooled results of active treatments

|  | Placebo (n = 16) | | |  | | ET + EPT (n = 35) | | | |  | |
| --- | --- | --- | --- | --- | --- | --- | --- | --- | --- | --- | --- |
|  | Baseline 12 weeks | | | | Change from  baseline | | Baseline 12 weeks | | Change from  baseline | |  |
| Daytime measurement | | |  | |  | |  |  |  | |  |
| SBP | 147.9 ± 14.3 | | 146.9 ± 11.0 | | -0.9 ± 12.5 | | 148.9 ± 13.0 | 147.6 ± 16.2 | -1.4 ± 11.9 | |  |
| DBP | 92.8 ± 9.4 | | 91.0 ± 10.6 | | -2.3 ± 8.7 | | 94.0 ± 10.0 | 93.6 ± 10.6 | -0.4 ± 6.6 | |  |
| HR | 72.8 ± 6.8 | | 72.0 ± 7.4 | | 0.1 ± 6.4 | | 75.1 ± 7.1 | 73.1 ± 7.1 | -2.0 ± 6.0 | |  |
| BP load^a^ | | |  | |  | |  |  |  | |  |
| SBP | 77.0 ± 26.0 | | 74.1 ± 25.5 | | -2.4 ± 28.2 | | 75.5 ± 24.6 | 70.4 ± 26.6 | -5.1 ± 23.7 | |  |
| DBP | 74.3 ± 25.5 | | 66.2 ± 30.9 | | -10.5 ± 23.7 | | 68.7 ± 25.1 | 68.1 ± 29.0 | -0.5 ± 18.9 | |  |
| Nighttime measurement | | | | |  | |  |  |  | |  |
| SBP | 139.9 ± 16.8 | | 136.4 ± 12.6 | | -3.7 ± 12.4 | | 140.0 ± 13.7 | 139.8 ± 15.5 | -0.2 ± 13.5 | |  |
| DBP | 85.3 ± 11.5 | | 82.4 ± 9.4 | | -3.2 ± 7.8 | | 86.2 ± 10.9 | 84.8 ± 10.2 | -1.4 ± 6.9 | |  |
| HR | 64.0 ± 6.4 | | 62.2 ± 6.6 | | -1.2 ± 7.8 | | 63.7 ± 6.8 | 62.1 ± 5.4 | -1.6 ± 5.8 | |  |
| BP load^a^ | | |  | |  | |  |  |  | |  |
| SBP | 79.1 ± 25.9 | | 77.8 ± 28.5 | | -2.2 ± 32.1 | | 79.8 ± 25.3 | 74.5 ± 29.8 | -5.3 ± 32.1 | |  |
| DBP | 59.9 ± 27.9 | | 49.4 ± 32.6 | | --10.8 ± 28.3 | | 61.6 ± 34.4 | 56.5 ± 36.0 | -5.2 ± 32.4 | |  |
| Night dip^b^ | | |  | |  | |  |  |  | |  |
| SBP | -5.3 ± 7.4 | | -7.8 ± 7.0 | | -2.7 ± 6.3 | | -5.7 ± 8.8 | -5.1 ± 6.0 | 0.6 ± 6.8 | |  |
| DBP | -8.0 ± 7.9 | | -9.6 ± 8.0 | | -1.4 ± 7.5 | | -8.0 ± 9.6 | -9.0 ± 8.6 | -1.0 ± 8.0 | |  |
| 24-hour measurement | | |  | |  | |  |  |  | |  |
| SBP | 146.0 ± 14.1 | | 144.4 ± 10.5 | | -1.5 ± 11.7 | | 146.7 ± 11.9 | 145.5 ± 15.6 | -1.2 ± 11.4 | |  |
| DBP | 91.0 ± 9.4 | 89.0 ± 9.9 | | | -2.5 ± 7.9 | | 92.2 ± 9.4 | 91.4 ± 9.8 | -0.8 ± 5.7 | |  |

Data are presented as means ± standard deviations

ET estrogen therapy, EPT estrogen + progestogen therapy, SBP systolic blood pressure (mmHg), DBP diastolic blood pressure (mmHg), HR heart rate (bpm), BP blood pressure

^a^ proportion (%) of BP higher than the predefined cutoff level (135/85 mmHg for the daytime and 130/80 mmHg for the nighttime)

^b^BP difference (mmHg) between the daytime and nighttime

There was no difference in change from baseline either between the two groups or within the group.
